# Supplementary material for: An allosteric modulator activates BK channels by perturbing coupling between Ca2+ binding and pore opening
Source: Nat Commun. 2022 Nov 9;13:6784. doi: 10.1038/s41467-022-34359-6 (PMC9646747; doi:10.1038/s41467-022-34359-6)
Supplement: Supplementary file 1 — Supplementary Information [file 41467_2022_34359_MOESM1_ESM.pdf]

Supplementary Information

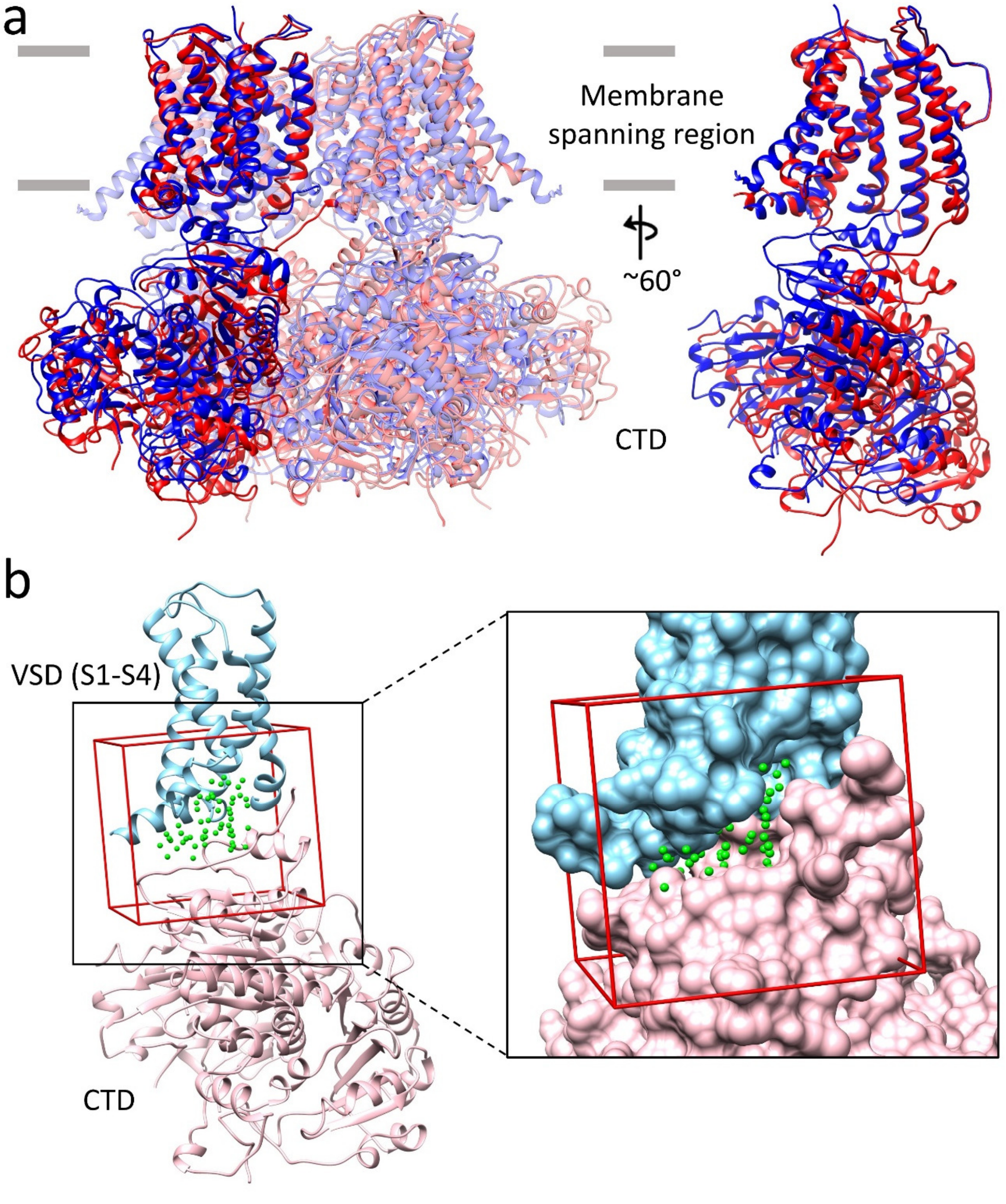

Supplementary Figure 1. a, Structural comparison between open (colored blue, PDB: 6v38) and closed (colored red, PDB: 6v3g) conformations of the human BK channel. The membrane spanning region (S0-S6) of the two conformations were superimposed by UCSF Chimera[1]. In the right panel, the membrane spanning region and CTD are from two neighboring subunits. **b,** The screening site locates at the VSD-CTD interface (in the cubic box). The VSD is colored cyan and the CTD is colored pink. The sphere points (colored green) were generated from UCSF DOCK[2] (http://dock.compbio.ucsf.edu/), representing a negative image of the protein binding surface to be matched with the atomic centers of the compounds in the screening database.

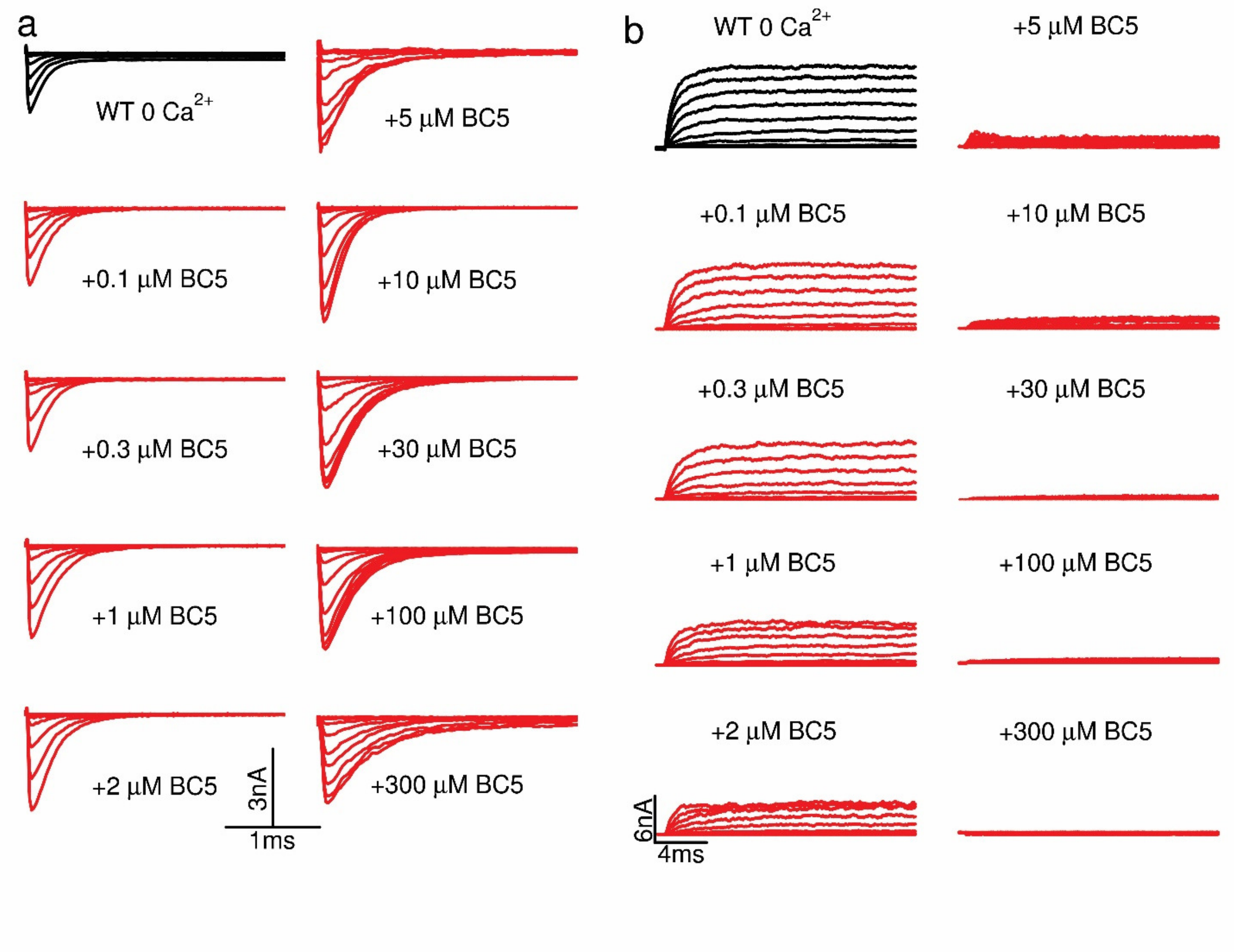

Supplementary Figure 2. Current traces for BK channels at various BC5 concentrations. **a,** Tail currents. All of tail currents were collected at -80 mV after testing pulses from -80 mV to 200 mV at 20 mV increase. Note that the tail currents started at more negative testing pulses with increasing BC5 concentrations, corresponding to the GV shift in response to BC5 (Fig. 1d). **b,** Current traces in response to testing pulses from -80 mV to 200 mV at 20 mV increment. The current amplitudes at +250 mV were used to calculate dose response of BC5 inhibition (Fig. 1e).

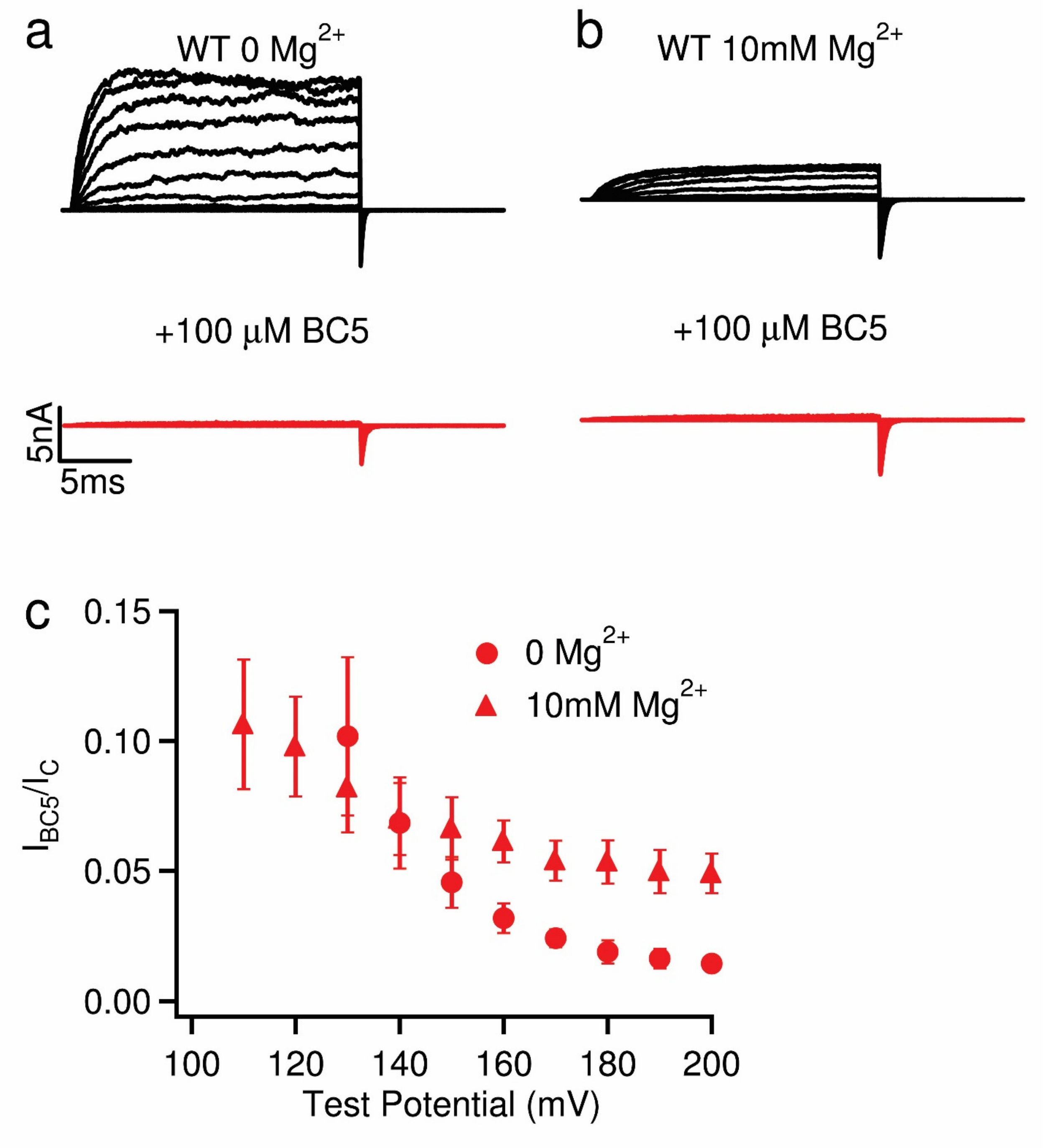

Supplementary Figure 3. BC5 inhibition of BK channels with and without 10 mM Mg^{2+}. **a,** Current traces in 0 Mg^{2+}. Voltage pulses: -30mV to 250mV for control (black), -80mV to 200mV in 100 µM BC5 (red), at 20mV increment. The potentials before and after testing pulses were -80 mV. **b,** Current traces in 10 mM Mg^{2+}. Voltage pulses were the same as in A except for from -

80mV to 200mV. **c,** The ratio of steady state outward current at 100 μM BC5 compared to control at various voltages with (filled triangles, n=3) or without (filled circles, n=3) the presence of 10 mM Mg^{2+}. Note that the BC5 inhibited ≥95% BK currents at 200 mV either with or without 10 mM Mg^{2+}. In the presence of Mg^{2+} BC5 inhibition was reduced by a small percentage, possibly due to the interference between Mg^{2+} and BC5, which are both voltage dependent blockers of the channel.

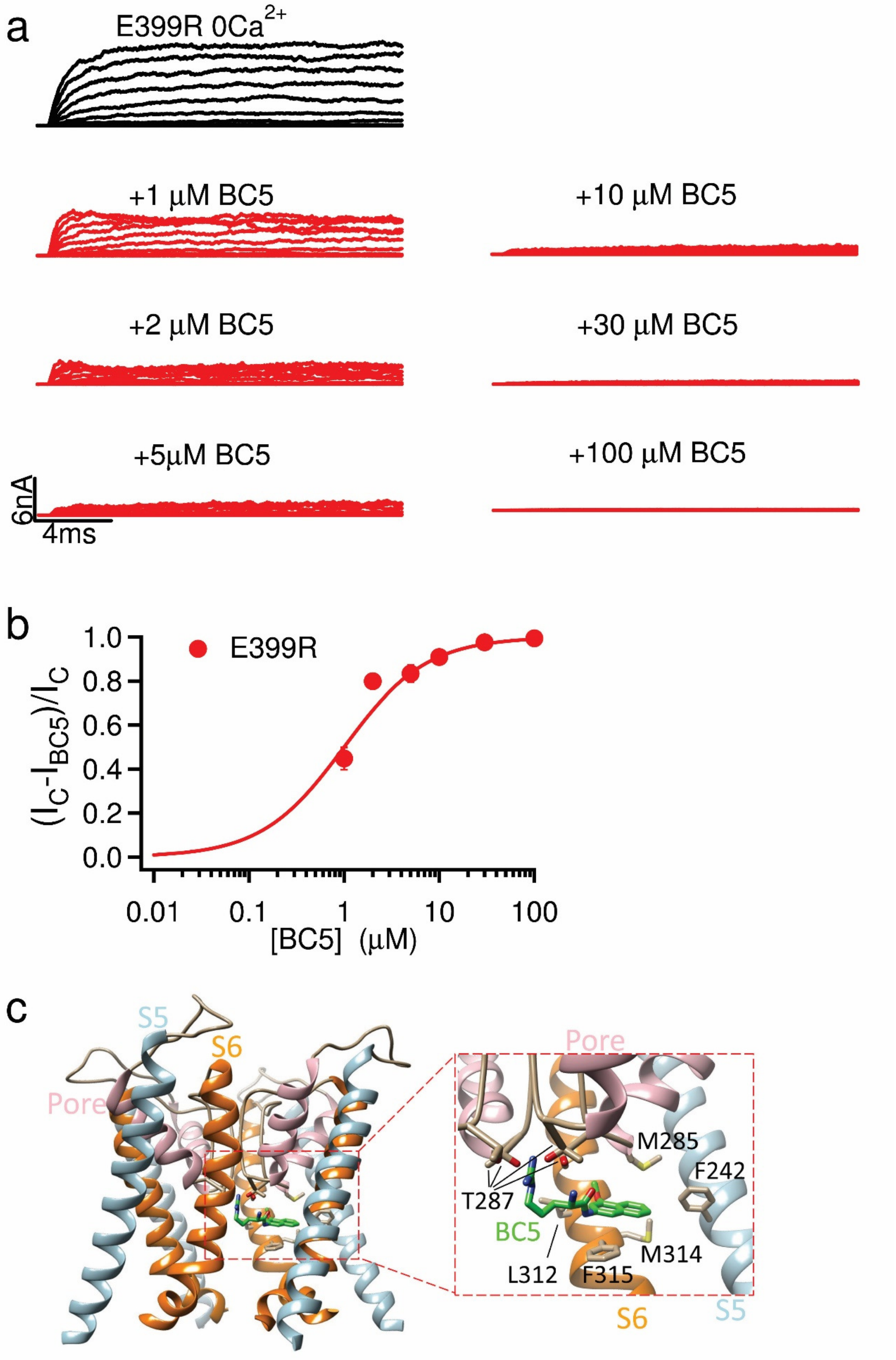

a
E399R 0Ca2+
+1 μM BC5
+10 μM BC5
+2 μM BC5
+30 μM BC5
+5μM BC5
+100 μM BC5
6nA
4ms
b
E399R
(IC-IBC5)/IC
1.0
0.8
0.6
0.4
0.2
0.0
0.01
0.1
1
10
100
[BC5] (μM)
c
S5
S6
Pore
Pore
M285
F242
T287
BC5
M314
L312
F315
S6
S5

Supplementary Figure 4. BC5 inhibition of a mutant BK channel with altered BC5 activation, and a possible mechanism of pore blocking. **a,** Current traces of BK channels with the mutation E399R at various BC5 concentrations. **b,** Current inhibition in various BC5 concentrations (n=3). Current traces at +200 mV were used to calculate the dose response curve with IC_{50} of 1.0 µM. Note that the mutation E399R reduced BC5 activation, increasing the EC_{50} of $V_{1/2}$ shift to more than 5 fold (Fig. 2f), but the mutation did not enhance IC_{50} of BC5 inhibition (Fig. 1e). **c,** BC5 docked in the pore of the BK channel (PDB 6v3g). S5, the pore helix and S6 are colored light blue, lavender and orange, respectively. BC5 is represented by the stick model and its carbon atoms are colored green. Residues interacting with BC5 are shown by the stick model and their carbon atoms are colored tan.

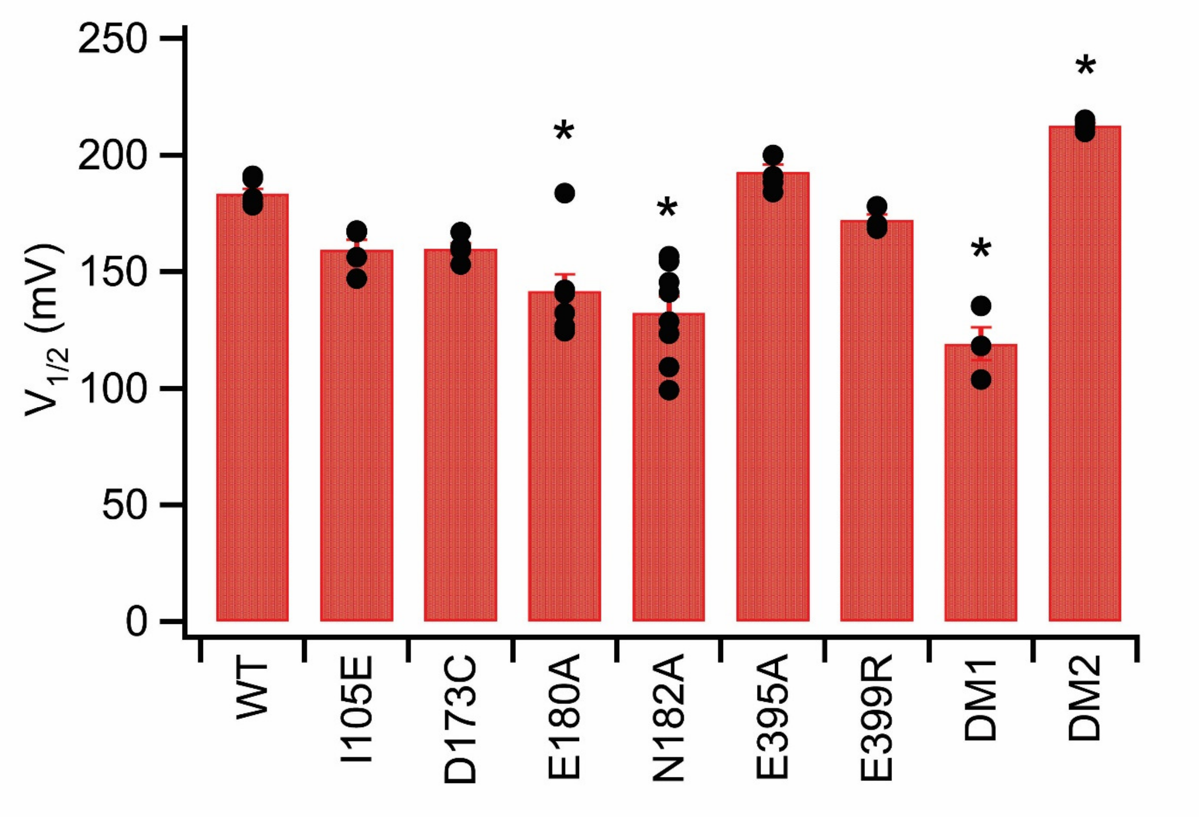

Supplementary Figure 5. $V_{1/2}$ of GV relationships at nomial 0 Ca^{2+} for the WT and mutant BK channels that are shown in Fig. 2e. Data points are shown in solid circles. *: compared to WT with P<0.05, one-way Tukey–Kramer ANOVA test. For WT, n=8; for I105E, n=4; for D173C, n=5; for E180A, n=6; for N182A, n=8; for E395A, n=5; for E399R, n=3; for DM1 (E180AN182A), n=3; for DM2 (I105EE399R), n=3.

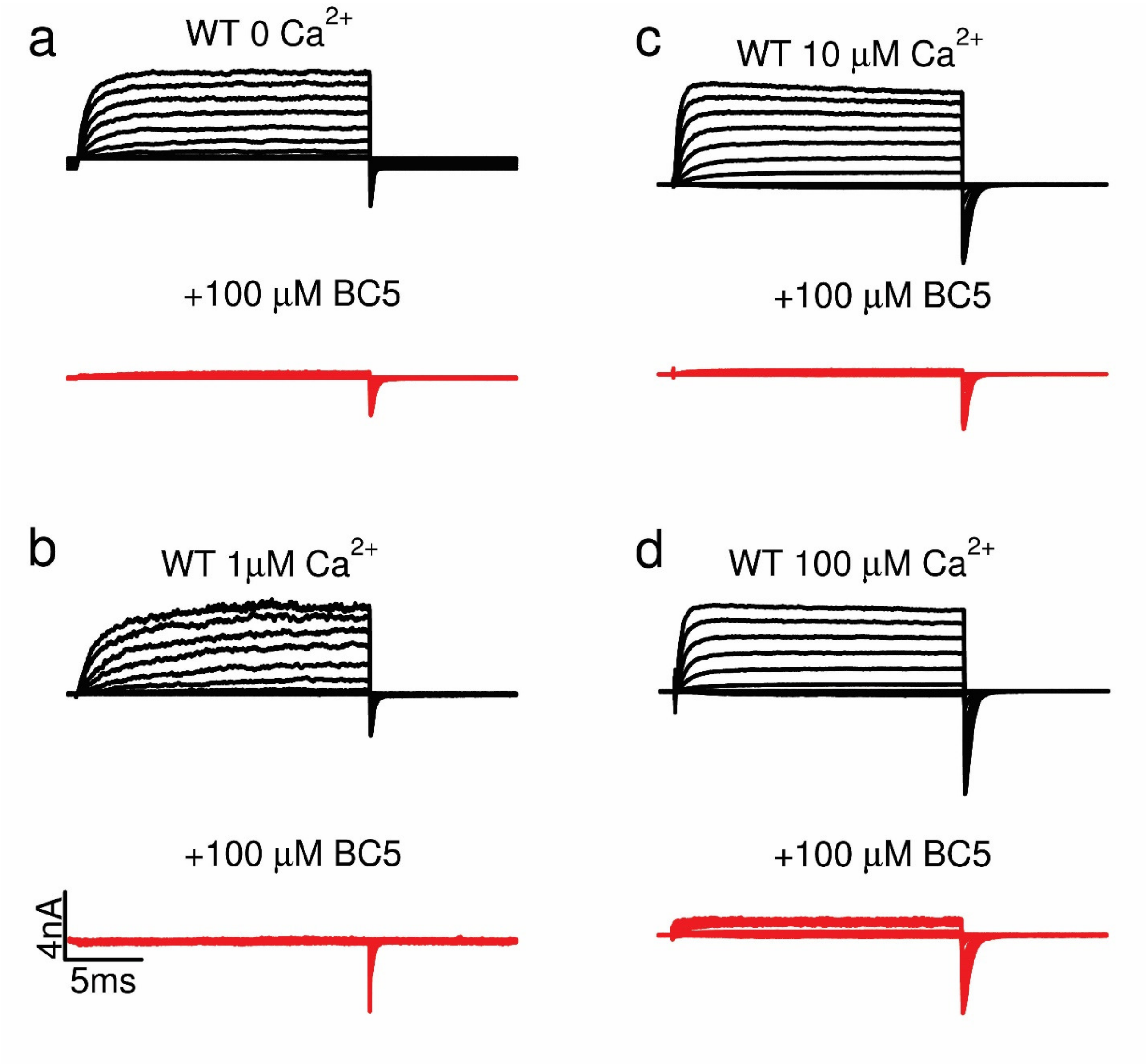

Supplementary Figure 6. a-d Current traces of BK channels in the presence of different Ca^{2+} concentrations and 100 µM BC5 (corresponding to GV relationships in Fig. 3c).

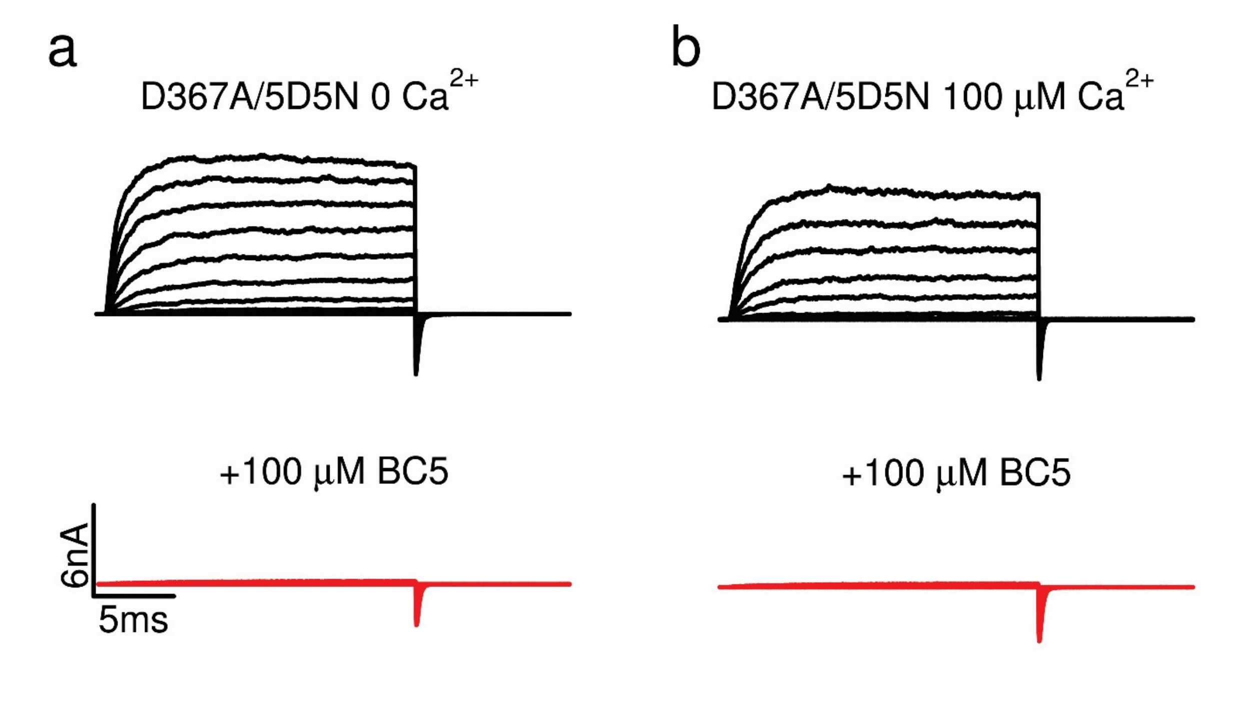

Supplementary Figure 7. Current traces of mutation D367A/5D5N that removed both Ca^{2+} binding sites of BK channels (corresponding to GV relations in Fig. 3e). **a,** In nominal 0 Ca^{2+}, with (red) and without (black) 100 µM BC5. **b,** In100 µM free Ca^{2+}.

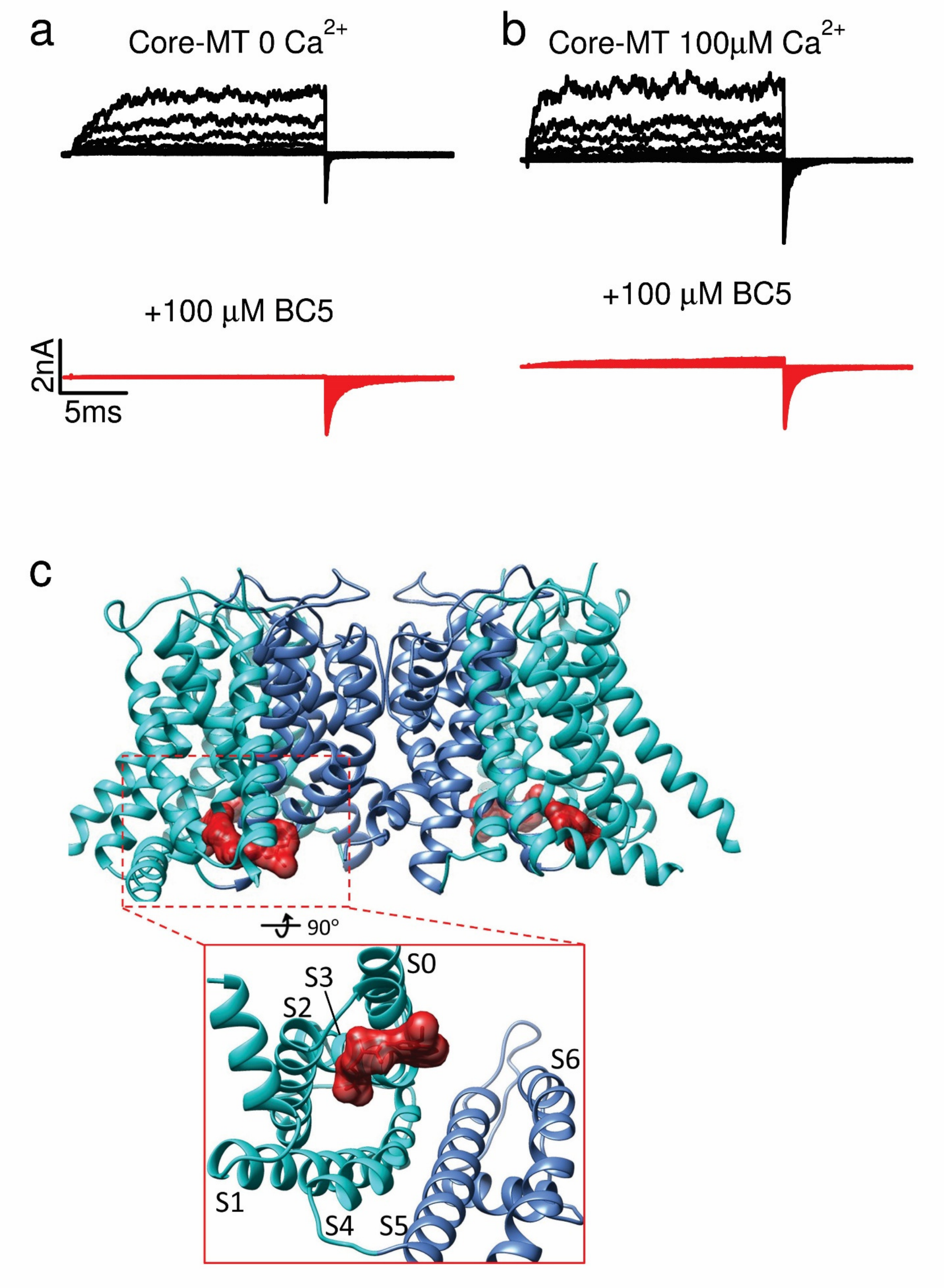
a
Core-MT 0 Ca^{2+}
b
Core-MT 100μM Ca^{2+}
+100 μM BC5
+100 μM BC5
2nA
5ms
c
90°
S0
S3
S2
S6
S1
S4
S5

Supplementary Figure 8. BC5 activation of the Core-MT BK channel. **a, b,** Current traces of Core-MT (corresponding to GV relations in Fig. 3f). **c,** Docking of BC5 on the core-MT (PDB 6v38). BC5 is represented by the surface model and colored red. The tetramer Core-MT is shown by the ribbon model. VSDs are colored cyan and pore-gate domains are colored cornflower blue.

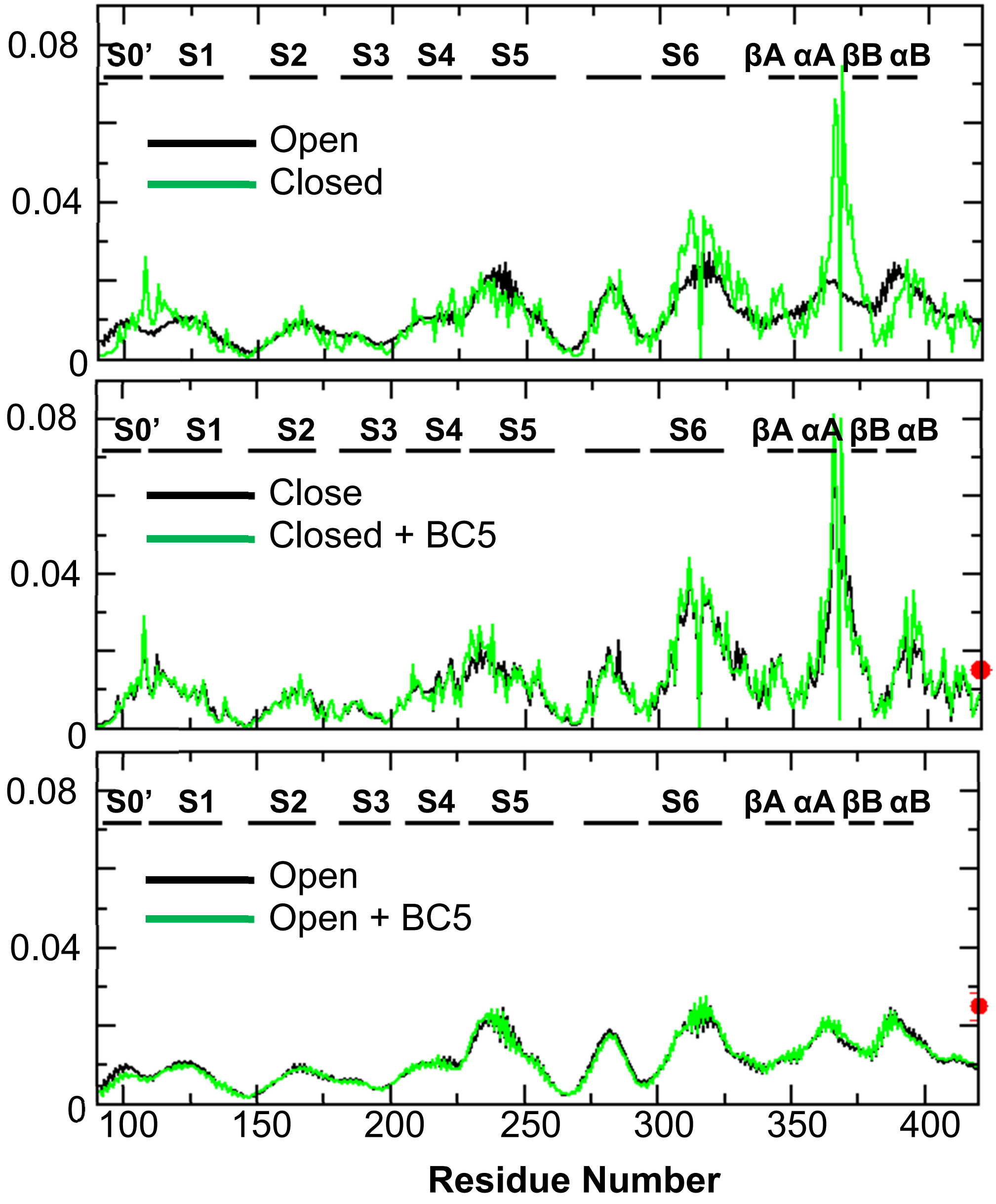

Supplementary Figure 9. Averaged information flow profiles of BK channels under various conditions. The source and sink nodes are D367 and F315, respectively. Protein specific domains are labeled above the plots. The information flow though BC5 is also shown as red circle, which is stronger in the open state.

.

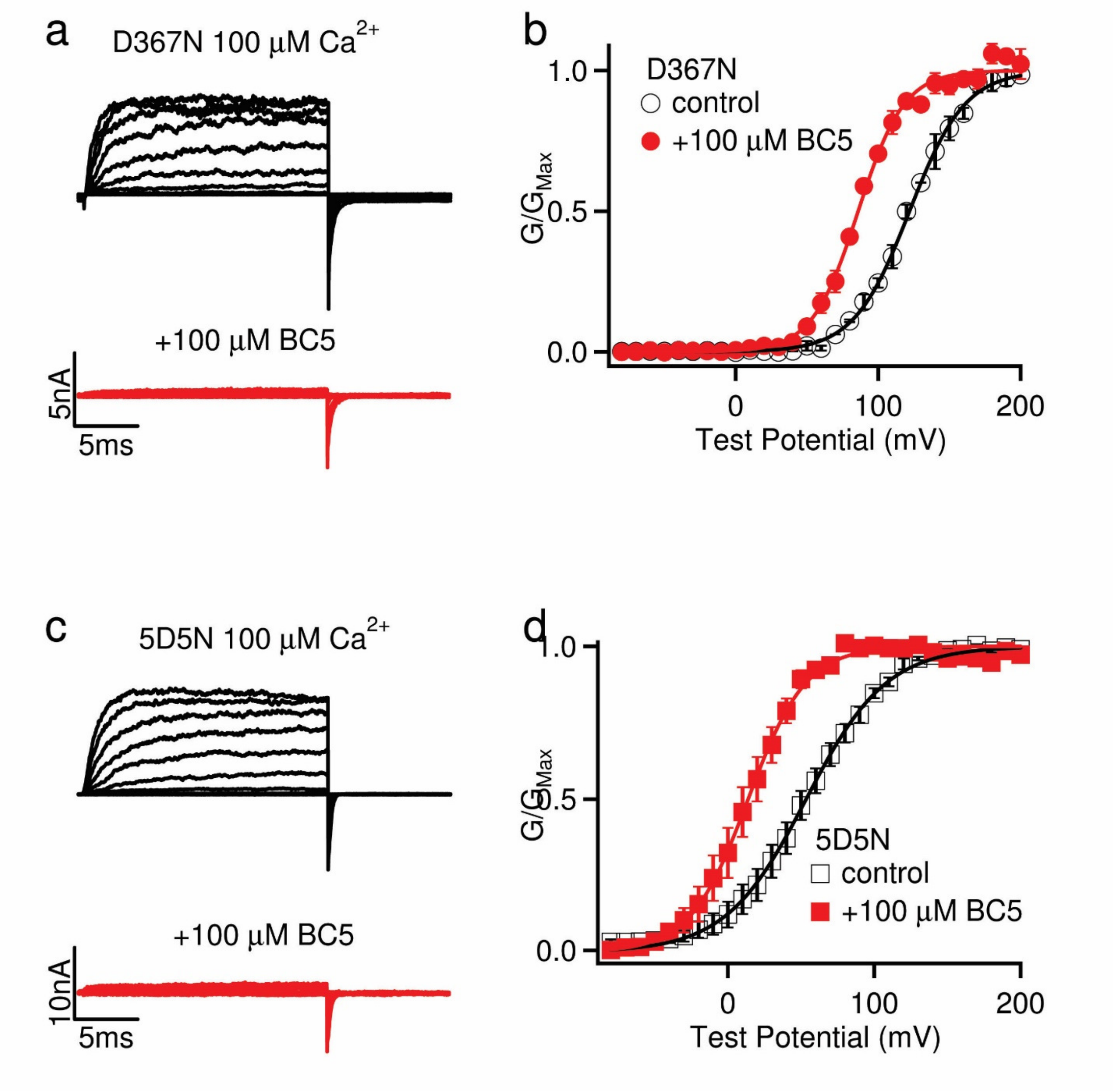

Supplementary Figure 10. BC5 effect in relation to different Ca^{2+} binding sites. **a,** Current traces in 100 μM $[Ca^{2+}]_i$ for mutation D367N, which ablated the Ca^{2+} binding site in RCK1 were elicited by various testing voltages from -80mV to 200mV with 20mV increment, control (black) and with BC5 (100 μM, red) . The potential before and after testing pulses was -80 mV. **b,** BC5 shifted the GV relation of the D367N mutant channel in 100 μM $[Ca^{2+}]_i$. GV relations were fit with the Boltzmann function (solid lines) with $V_{1/2}$ and slope factor (mV): 122.0 ± 3.2 and 19.5 ± 2.8 for

control (n=3); and 86.0 ± 3.9 and 16.2 ± 3.4 with 100 μM BC5 (n=3). **c,** Current traces for mutation 5D5N, which ablated the Ca^{2+} binding site in RCK2. Conditions and symbols are similar to a. **d,** BC5 shifted the GV relation of the 5D5N mutant channel in 100 μM $[Ca^{2+}]_i$. GV relations were fit with the Boltzmann function (solid lines) with $V_{1/2}$ and slope factor (mV): 53.4± 4.2 and 27.5 ± 3.7 for control (n=4); and 13.8 ± 4.4 and 19.8 ± 3.9 with 100 μM BC5 (n=4).

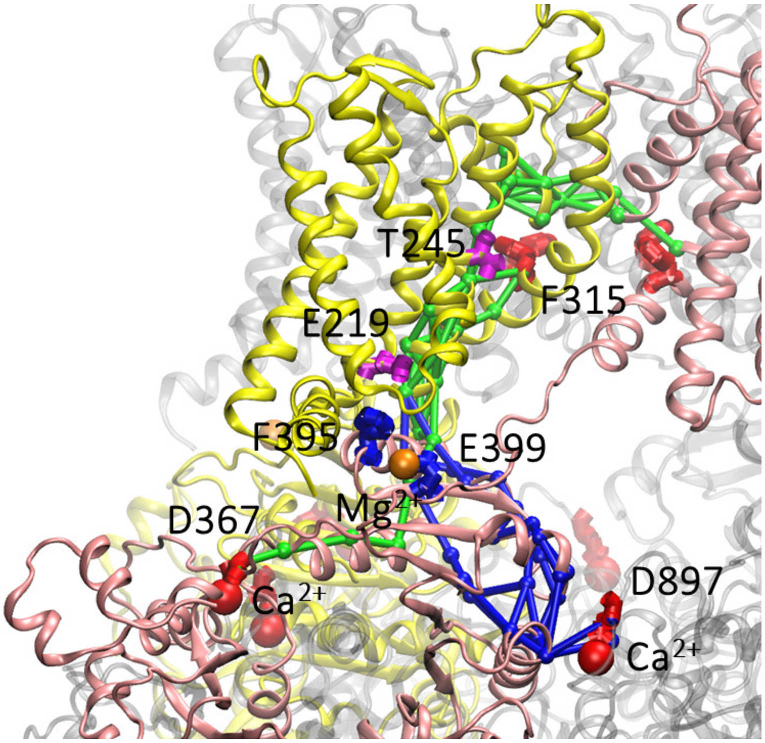

Supplementary Figure 11. Optimal and suboptimal pathways coupling Ca^{2+} binding site in RCK1 (D367) and RCK2 (D897) and pore lining residue F315. The two neighboring subunits forming the VSD-CTD contacts are colored in pink and yellow, respectively Residues F315 D367 D897 are colored in red, T245 E219 in purple and F395 E399 in blue. Bound Ca^{2+} ions are colored in red and Mg^{2+} in orange. The pathways from Ca^{2+} binding site in RCK1 (D367) and RCK2 (D897) are represented in green and blue sticks, respectively. The pathways from the two Ca^{2+} binding sites overlap in the membrane -spanning domain and are represented in green sticks.

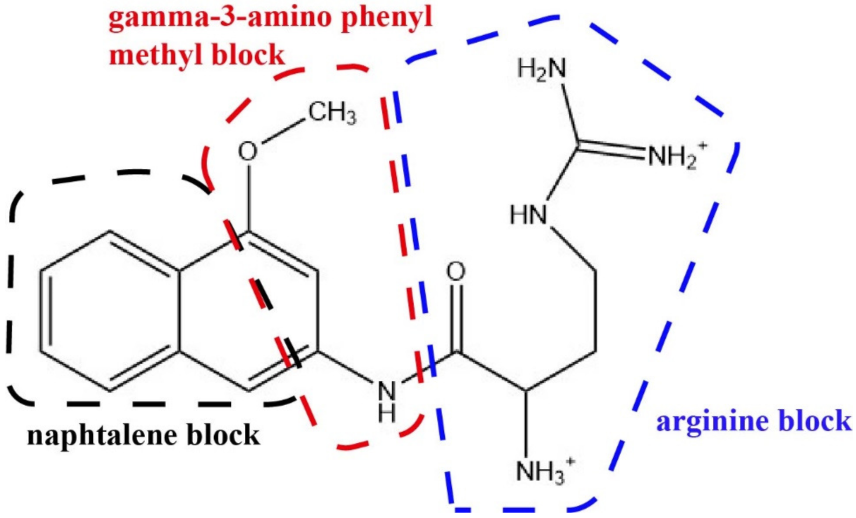

Supplementary Figure 12. Chemical building blocks used for deriving the BC5 topology. Naphtalene (Charmn36cgenff), gamma-3-amino phenyl (Charmn36cgenff) and arginine (CHARMM36m) blocks were chosen and assembled to generate the BC5 topology in both charm and gromacs format.

Supplementary Table 1 Summary of atomistic simulations of BK channels with and without BC5.

Systems	Ca^{2+}/Mg^{2+}	BC5	Initial Structure	Length (ns)
sim 1-3	+/-	+	6v38	200 each
sim 4-6	+/+	-	6v38	200 each
sim 7-9	-/-	+	6v3g	200 each
sim 10-12	-/-	-	6v3g	200 each

Supplementary Table 2 BK residues with >40% probability of contacting BC5 in the open or closed state of the channel.

			BC5 Contact Probability		Interaction Type
**Domain**	**Region**	**Residue**	**Closed***	**Open***	
VSD	S0	K 98	0.01±0.003	0.43±0.04	H-bond
		G 102	0.21±0.09	0.44±0.07	N/A
		I 105	0.46±0.25	0.92±0.09	hydrophobic
	S1	A 107	0.46±0.17	0.20±0.01	hydrophobic
		L 166	0.02±0.01	0.52±0.04	N/A
		R 167	0.29±0.11	0.92±0.10	cation-π
		F 168	0.07±0.06	0.67±0.12	N/A
		A 170	0.23±0.11	0.95±0.06	hydrophobic
	S3	A 171	0.16±0.06	0.95±0.06	hydrophobic
		N 172	0.22±0.07	0.95±0.04	H-bond
		D 173	0.16±0.06	0.80±0.17	salt bridge
		F 177	0.15±0.10	0.87±0.09	π-π
		E 180	0.28±0.02	0.70±0.04	salt bridge
	S4	Q 216	0.41±0.23	0.11±0.05	H-bond
		E 219	0.49±0.21	0.27±0.04	salt bridge
		F 223	0.42±0.14	0.12±0.15	π-π
C-linker		K 331	0.07±0.07	0.47±0.08	H-bond
		G 333	0.11±0.12	0.42±0.20	H-bond
		S 335	0.09±0.07	0.59±0.02	H-bond
		Y 336	0.39±0.13	0.78±0.15	H-bond
		S 337	0.43±0.12	0.72±0.09	H-bond
		V 339	0.44±0.14	0.39±0.11	hydrophobic
		R 342	0.30±0.20	0.46±0.12	N/A
CTD	βB	E 374	0.43±0.16	0.71±0.13	salt bridge
	αB	F 395	0.32±0.17	0.98±0.01	π-π
		T 396	0.42±0.18	0.74±0.07	H-bond
	βC	Q 397	0.29±0.19	0.53±0.07	H-bond
		V 398	0.20±0.15	0.61±0.15	H-bond
		E 399	0.41±0.13	0.98±0.02	salt bridge
		Y 401	0.20±0.08	0.87±0.10	cation-π

* The data is obtained by average over the time-averaged results of three simulations (*sim 1-3* for open state and *sim 7-9* for closed state). The standard error between three simulations is also represented after the ± of each data.

Supplementary Table 3 Nine compounds selected from *in silico* screening that were assayed, with their chemical names, structures, molecular weights, and computational energy scores (ITScore2).

ID	MDL	Chemical name	Chemical structure	Molecular weight	Vendor/ Catalog number	ITScore2
1	MFCD00051770	FMOC-L-Arginine	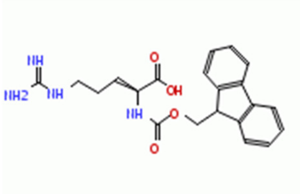	396.44	MilliporeSigma/ 47589	-44.1
2	MFCD01632025	Fmoc-(2s,6s,9s)-6-amino-2-carboxymethyl-3,8-diazabicyclo-(4,3,0)-nonane-1,4-dione	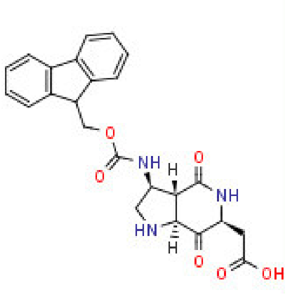	449.5	MilliporeSigma/ CH6371378872	-43.0
3	MFCD03939910	L-ALANYLGLYCYLGLYCYLGLYCINE	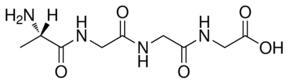	260.252	MilliporeSigma/ S777765	-41.1
4	MFCD00134470	3-[5-(Sulfophenyl)-2-pyridyl]-1,2,4-triazin-5-ylbenzenesulfonic acid disodium salt	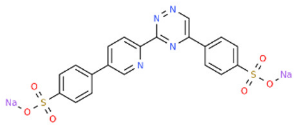	514.44	Ambinter/ Amb9809752	-38.0

5	MFCD00038393	ARG-4-METHOXY-2-NAPHTHYLAMINE	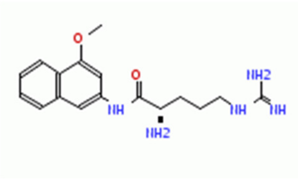	329.402	MP Biomedicals/ 03MNA002<br><br>or<br><br>MOlPort/ MolPort-046-683-309	-37.1
6	MFCD00046373	N-naphthalen-1-ylimidodicarbonimidicdiamide	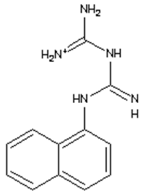	227.27	Vitas-M Lab/ STK133999	-37.0
7	MFCD00066054	Gly-Gly-Phe-OH	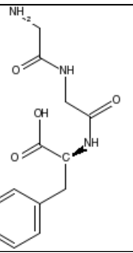	279.3	MilliporeSigma/ CH6371380198	-36.9
8	MFCD00179803	1-carbamimidamido-N-(3-ethynylphenyl)methanimidamide hydrochloride	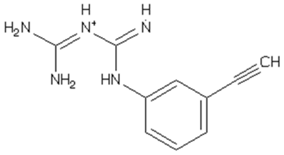	237.69	MolPort/ MolPort-002-916-389	-35.1
9	MFCD00051217	L-Glutamic acid alpha-(7-amido-4-methylcoumarin)	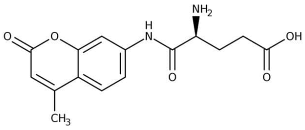	304.302	Fisher Scientific/ AAJ64847MF	-33.6

SUPPLEMENTARY REFERENCES

1 Pettersen, E. F. *et al.* UCSF Chimera--a visualization system for exploratory research and analysis. *Journal of computational chemistry* **25**, 1605-1612, doi:10.1002/jcc.20084 (2004).

2 Connolly, M. L. Analytical molecular surface calculation. *Journal of Applied Crystallography* **16**, 548-558, doi:https://doi.org/10.1107/S0021889883010985 (1983).
